# Supplementary material for: circMSH3 is a potential biomarker for the diagnosis of colorectal cancer and affects the distant metastasis of colorectal cancer
Source: PeerJ. 2023 Nov 7;11:e16297. doi: 10.7717/peerj.16297 (PMC10637257; doi:10.7717/peerj.16297)

**Fig 2 B**

The photo below corresponds to Figure 2B. The position indicated by the red line in the figure is the electrophoresis band of the qPCR product of circMSH3 in this article.

tagged photo


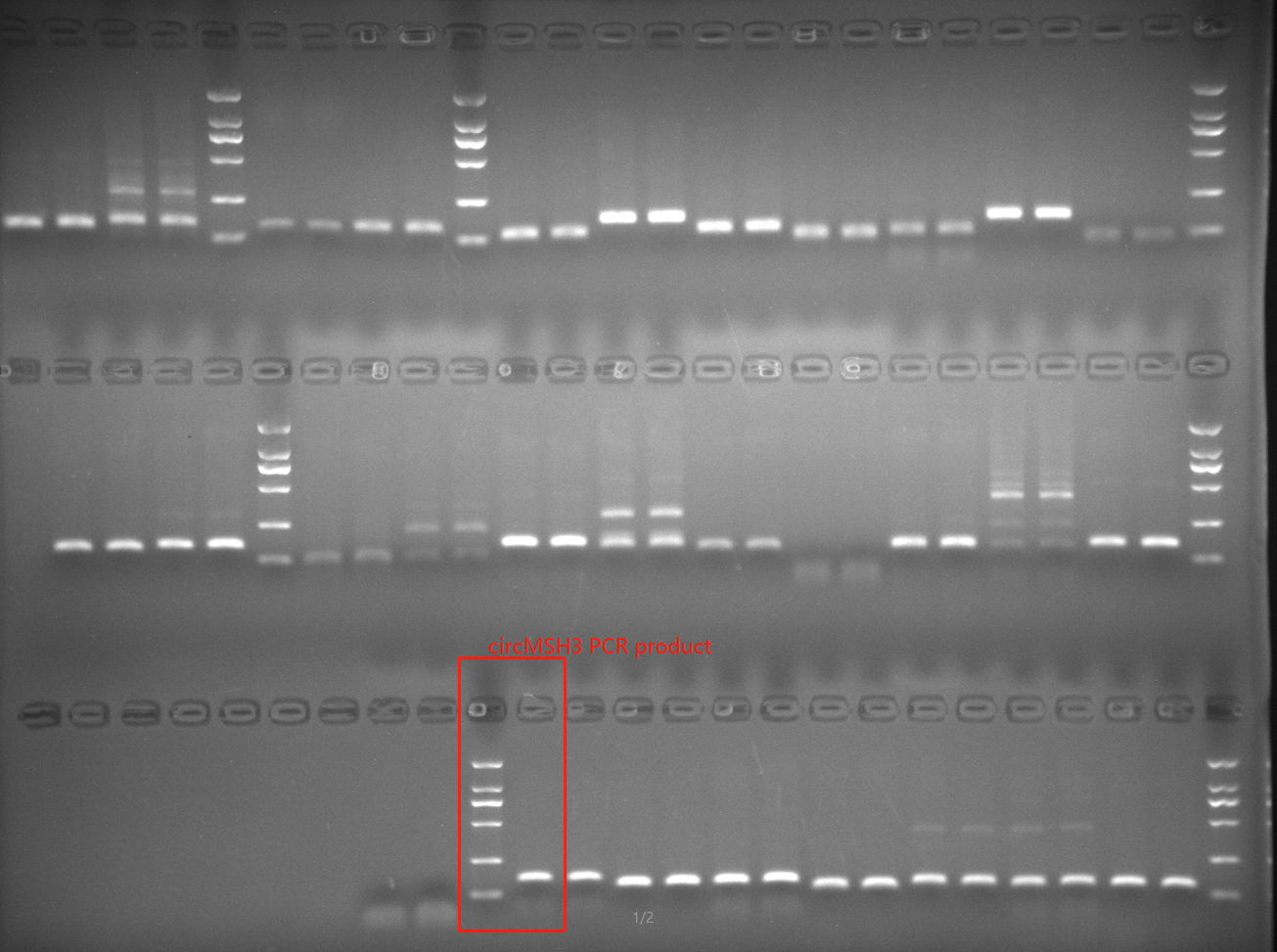


raw photo


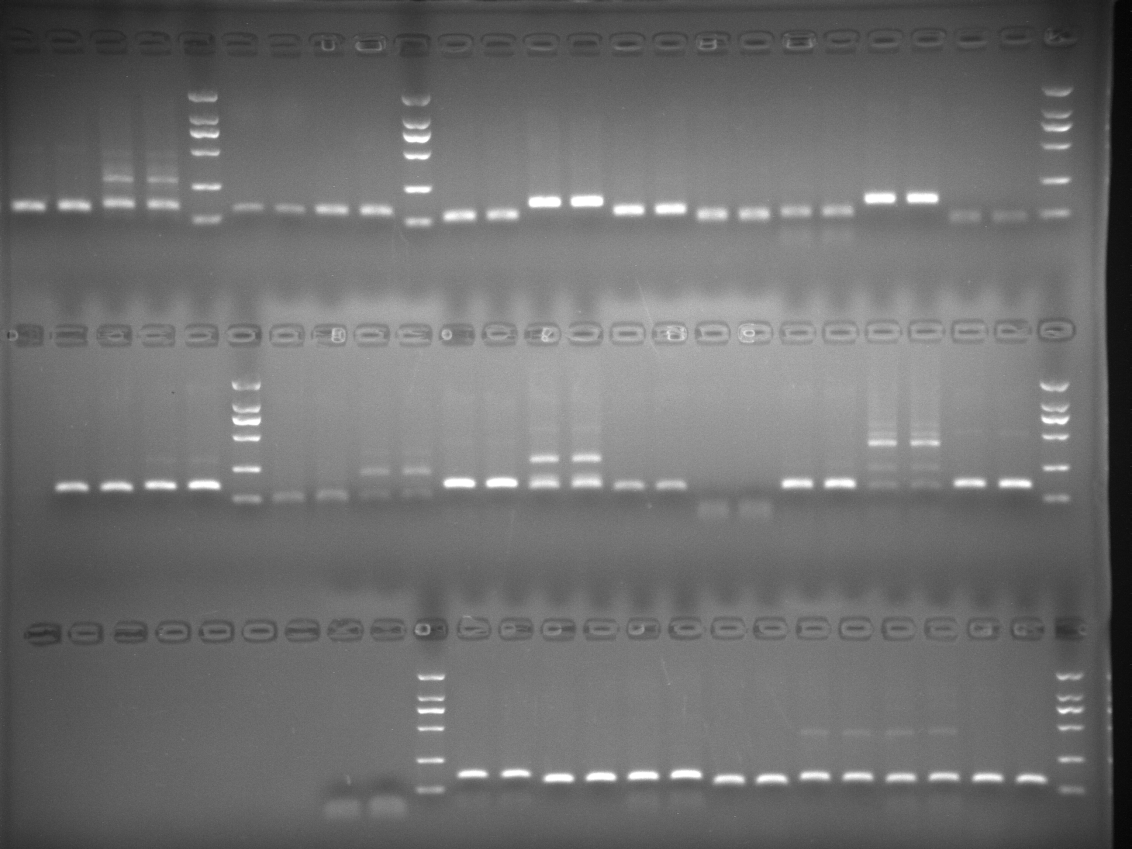


**Fig 4 C**

The photo below corresponds to the WB original film photo and marker in Figure 4c


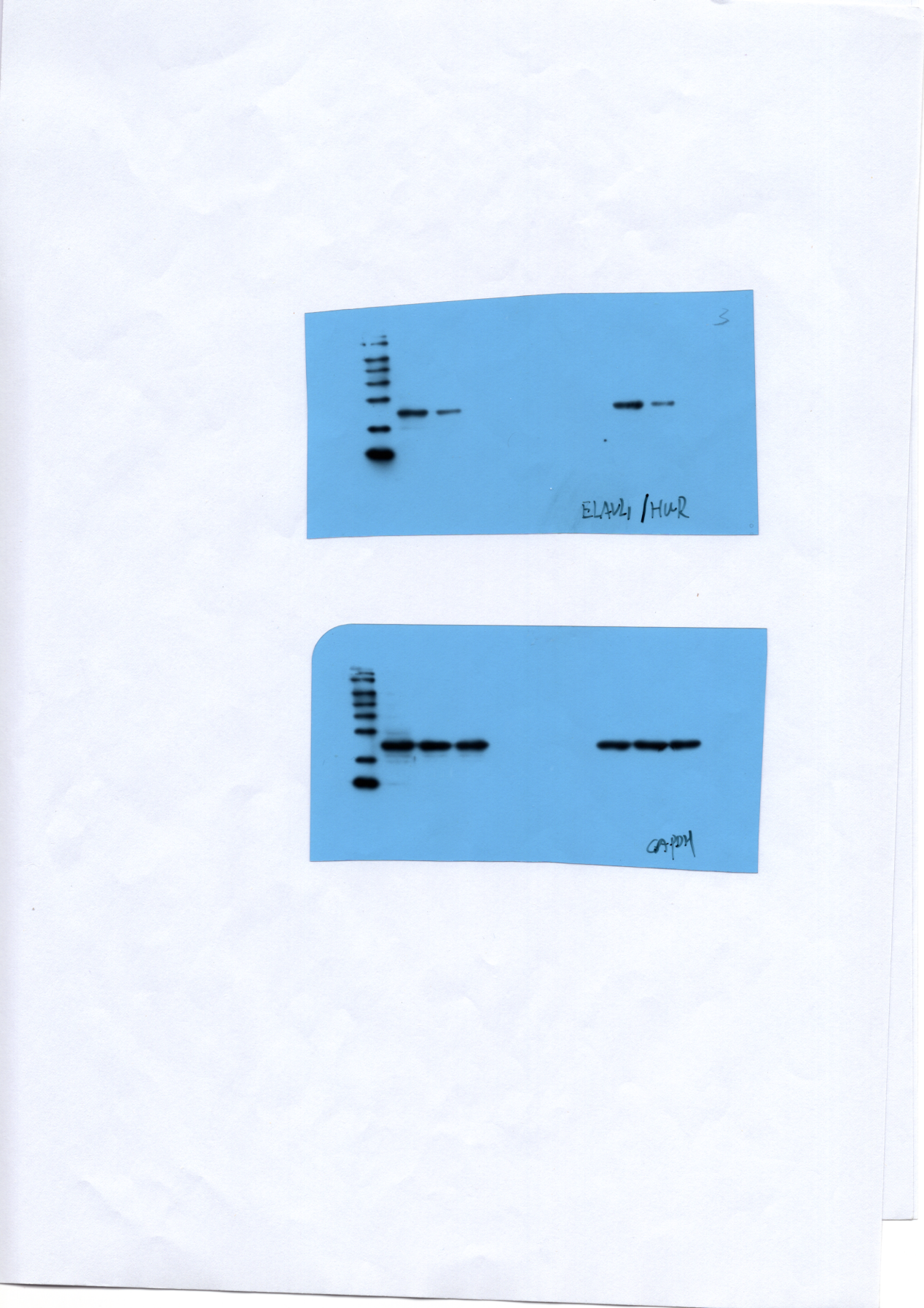


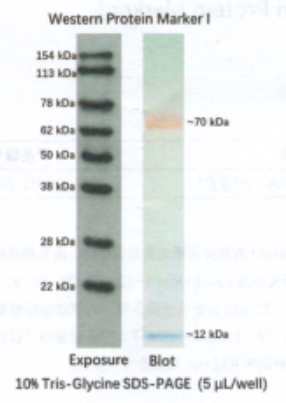


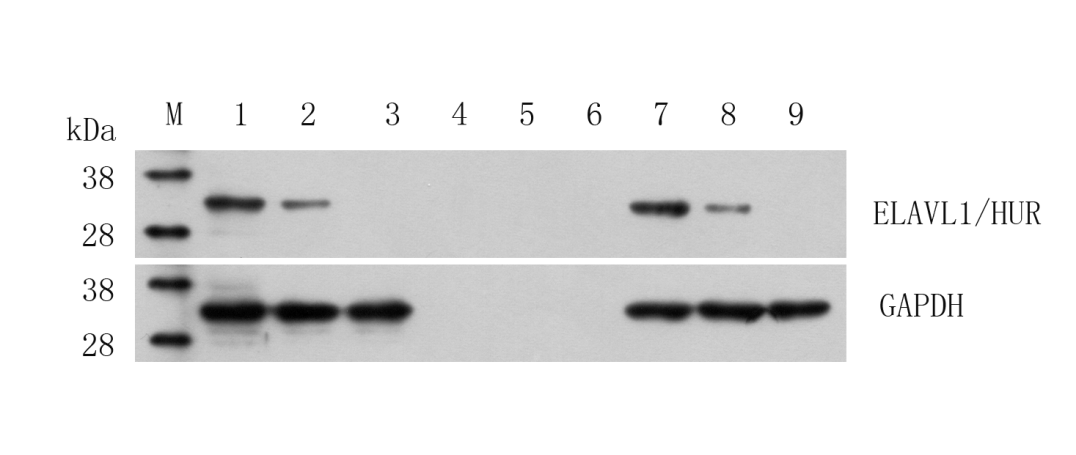

Supplement: Supplemental Information 2 — Fig. 2B corresponds to the original photo of qPCR product electrophoresis gel electrophoresis of circMSH3 [file peerj-11-16297-s002.docx]
