# Supplementary material for: circMSH3 is a potential biomarker for the diagnosis of colorectal cancer and affects the distant metastasis of colorectal cancer
Source: PeerJ. 2023 Nov 7;11:e16297. doi: 10.7717/peerj.16297 (PMC10637257; doi:10.7717/peerj.16297)

**Fig 4 C**

The photo below corresponds to the WB original film photo and marker in Figure 4c


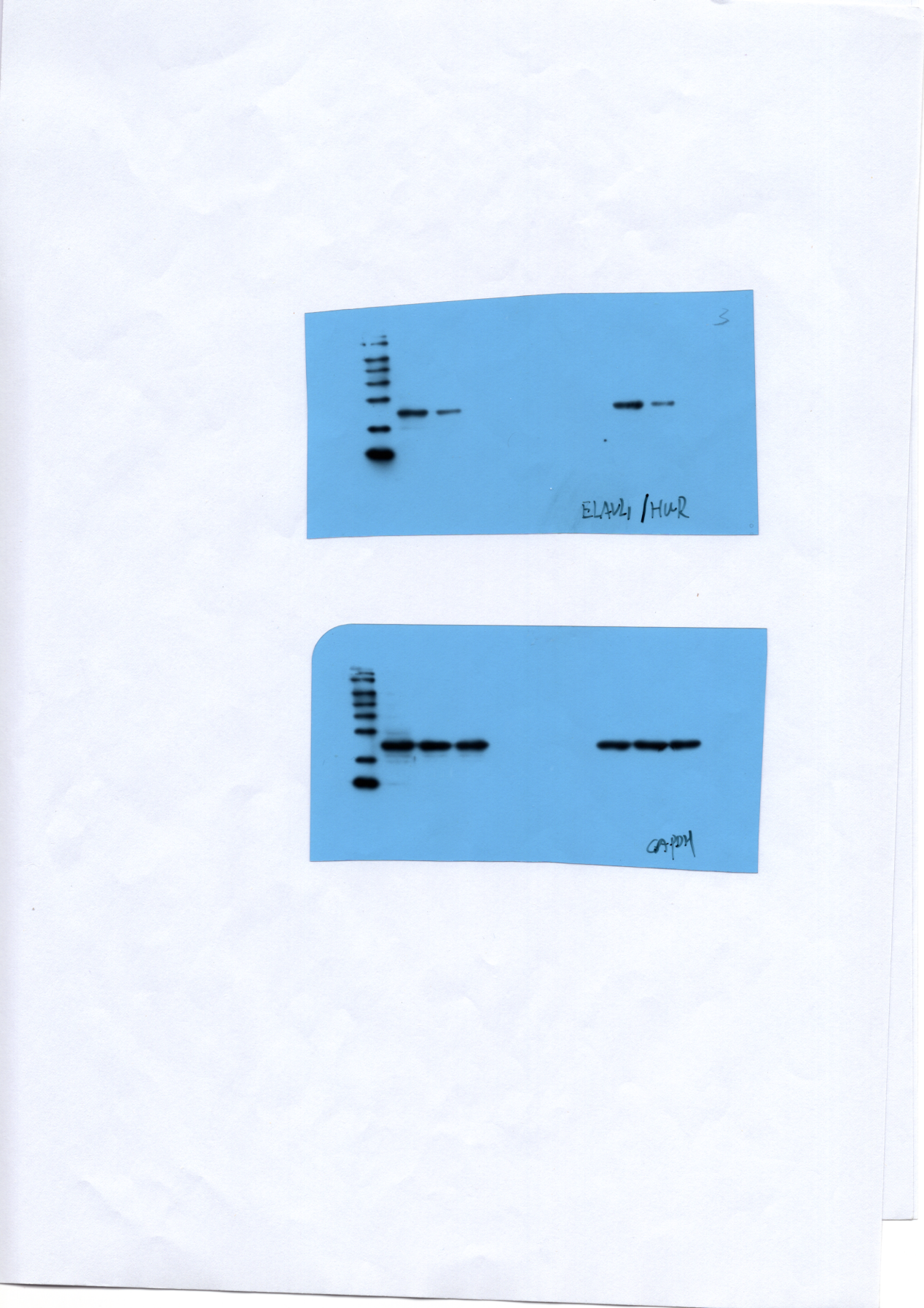


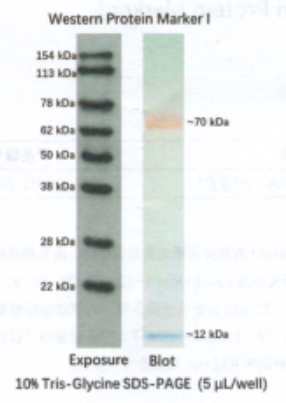


Below is the cropped image


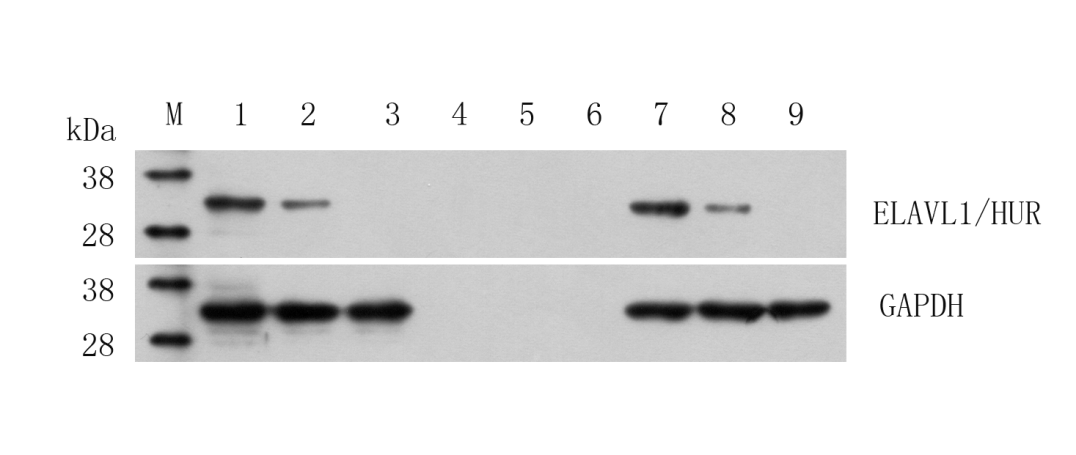

Supplement: Supplemental Information 3 — The photo of the original film corresponding to the WB electrophoresis results of the positive control HuR in the pulldown experiment [file peerj-11-16297-s003.docx]
